# High NOTCH1 mRNA Expression Is Associated with Better Survival in HNSCC

**DOI:** 10.3390/ijms19030830

**Published:** 2018-03-13

**Authors:** Markus Wirth, Daniel Jira, Armin Ott, Guido Piontek, Anja Pickhard

**Affiliations:** 1 Department of Otolaryngology-Head and Neck Surgery, Technical University of Munich, Ismaninger Straße 22, 81675 Muenchen, Germany; daniel.jira@web.de (D.J.); piontek@lrz.tu-muenchen.de (G.P.); a.pickhard@lrz.tu-muenchen.de (A.P.); 2 Institute of Medical Informatics Statistics and Epidemiology, Technical University of Munich, Ismaninger Straße 22, 81675 Muenchen, Germany; armin.ott@tum.de

**Keywords:** NOTCH signaling, HNSCC, overall survival

## Abstract

The clinical impact of the expression of NOTCH1 signaling components in squamous cell carcinoma of the pharynx and larynx has only been evaluated in subgroups. The aim of this study was therefore to evaluate NOTCH1 expression in head and neck squamous cell cancer (HNSCC) patient tissue and cell lines. We analyzed tissue from 195 HNSCCs and tissue from 30 normal patients for mRNA expression of NOTCH1, NOTCH3, HES1, HEY1, and JAG1 using quantitative real-time PCR. Association of expression results and clinical orpathological factors was examined with multivariate Cox regression. NOTCH1 expression was determined in three Human Papilloma Virus (HPV)-positive and nine HPV-negative HNSCC cell lines. High expression of NOTCH1 was associated with better overall survival (*p* = 0.013) and disease-free survival (*p* = 0.040). Multivariate Cox regression confirmed the significant influence of NOTCH1 expression on overall survival (*p* = 0.033) and disease-free survival (*p* = 0.029). A significant correlation was found between p16 staining and NOTCH1 mRNA expression (correlation coefficient 0.28; *p* = 0.01). NOTCH1 was expressed at higher levels in HPV-positive HNSCC cell lines compared with HPV-negative cell lines, which was not statistically significant (*p* = 0.068). We conclude that NOTCH1 expression is associated with overall survival, and that inhibition of NOTCH1 therefore seems less promising.

## 1. Introduction

Head and neck cancer is ranked as the seventh most frequent cause of cancer death in the world and squamous cell carcinoma comprises the most common subgroup [1]. Despite ongoing advances in surgery and in radio- and chemotherapy, five-year survival rates for head and neck squamous cell carcinoma (HNSCC) remain still in the order of 50% to 60% [1,2]. Besides Human Papilloma Virus (HPV) status, prediction of clinical outcome and therapy are still based on histopathological and clinical parameters [3,4]. Novel therapeutic targets and markers to stratify patients are therefore urgently needed.

The NOTCH signaling pathway is becoming increasingly relevant in diverse tumor entities including HNSCC [5,6]. NOTCH signaling plays an integral part in cell fate and development by controlling proliferation, differentiation, angiogenesis, and apoptosis [7]. Initiation of signaling is mediated through binding of the ligands Jagged or Delta-like resulting in the cleavage and release of NOTCH intracellular fragments (NOTCH-IC) [5,7,8,9]. Subsequently, NOTCH-IC are translocated to the nucleus and interact with RBPJ, a DNA-binding protein [5,7]. This leads to transcription of targets such as the MYC transcription factor and HES and HEY family proteins [5,7].

Alterations in NOTCH signaling have been described in several cancers and result in tumor promotion or suppression depending on the cancer entity and context [5]. The predominant function of NOTCH signaling remains controversial—it may act as an oncogene, tumor suppressor, or even have a bimodal role [10]. Frequent mutations of the NOTCH receptor family were detected in HNSCC and most likely result in loss of function of the receptors [10,11,12]. A high incidence of nonsynonymous mutations was identified in 43% of Chinese patients with oral squamous cell carcinoma (OSCC) and the occurrence of mutations was associated with poor overall survival [13,14]. The expression of NOTCH1 pathway genes, however, has only been studied in small patient cohorts or in subgroups of HNSCC with contradictory results.

Currently, the clinical relevance of transcriptional alterations in the NOTCH signaling pathway in HNSCC is not well understood. The aim of this study was therefore to evaluate the expression of key components of the NOTCH pathway with quantitative real-time PCR in a larger HNSCC collective and in normal tissue. Secondly, the association of the expression with clinical and pathological parameters was investigated. Moreover, NOTCH1 expression was also analyzed in HPV-positive and -negative HNSCC cell lines.

## 2. Results

The clinical and pathological characteristics of this cohort are depicted in Table 1. Significant associations between high NOTCH1 expression and nodal stage and p16 status were found.

### 2.1. NOTCH1 and 3 and HES1 Significantly Lower in Tumor

**Figure 1:**
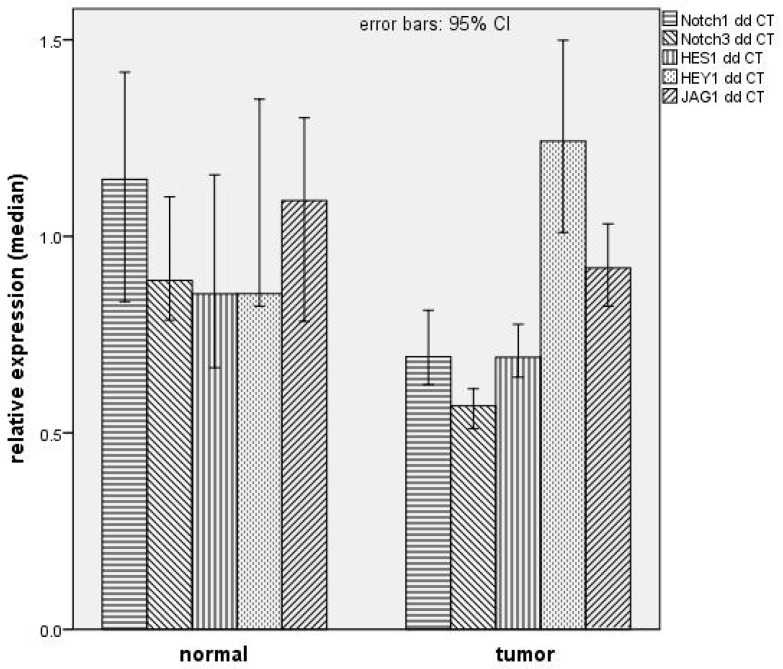
Depiction of median of relative mRNA expression in tumor tissue and normal tissue. Data were normalized to GAPDH per sample.

Relative mRNA expression of NOTCH1 and 3 and HES1 mRNA was significantly lower in tumor compared with in normal tissue (Table 2 and Figure 1). HEY1 mRNA was increased in HNSCC, but this was not significant (*p* = 0.254, Table 2). No significant difference between tumor tissue and normal tissue was detected for JAG1 mRNA expression (*p* = 0.270, Table 2).

### 2.2. High NOTCH1 Expression with Significant Longer Overall Survival

**Figure 2:**
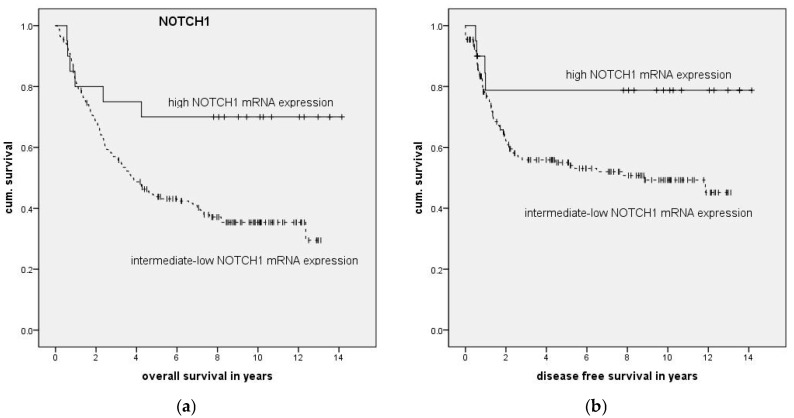
Association of relative NOTCH1 mRNA expression with overall (OS) and disease-free (DFS) survival was analyzed using the Kaplan-Meier method as well as the log-rank test. Patients were stratified into a relative high expression (mean mRNA expression + 1 standard deviation (SD)) group and an intermediate–low mRNA expression group (remainder). Significantly longer overall survival (**a**) and disease-free survival (**b**) were found for patients with relatively high mRNA expression of NOTCH1 compared with those with intermediate or low expression (*p* = 0.013 (OS) and *p* = 0.040 (DFS)).

In HNSCC patients, high mRNA expression of NOTCH1 was associated with better overall survival (OS, *p* = 0.013) and better disease-free survival (DFS, *p* = 0.040), as illustrated in Figure 2 and Table 3. Multivariate Cox regression confirmed the significant influence of high NOTCH1 expression on OS (*p* = 0.033) and DFS (*p* = 0.029) (Table 4).

Patients with low HEY1 mRNA expression demonstrated a significantly shortened disease-free survival (*p* = 0.040); this was also confirmed by multivariate Cox regression (*p* = 0.027, HR 1.69, Cox regression performed with clinical and pathological factors listed in Table 4 and low expression of NOTCH1 and HEY1). Prolonged DFS was seen in patients with high mRNA expression of downstream HEY1 (*p* = 0.077). Patients with low expression of NOTCH3 and downstream HES1 also showed a shortened DFS (*p* = 0.082 and *p* = 0.065). No significant alteration was observed for OS or DFS by JAG1 mRNA expression.

### 2.3. NOTCH1 Expression and p16 Staining

**Figure 3:**
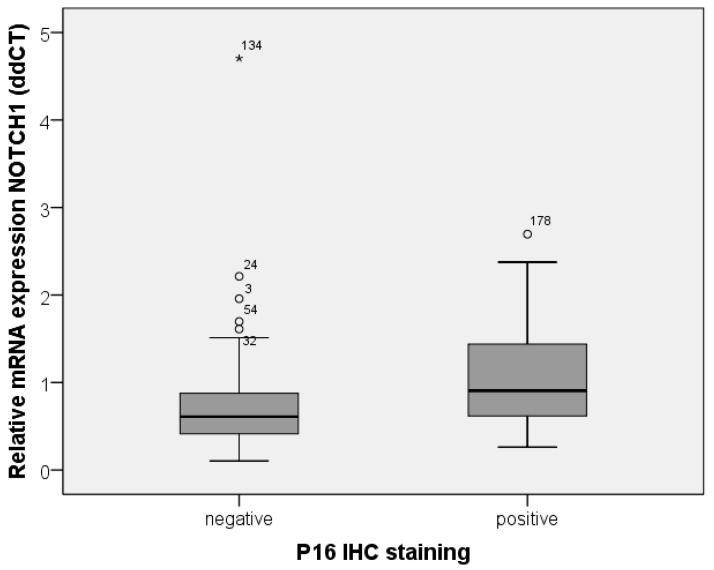
Depiction of median of relative mRNA expression of NOTCH1 in p16-positive and p16-negative tumor tissue. Expression of NOTCH1 was significantly higher in p16-positive tumor tissue (*p* ≤ 0.001, Mann–Whitney U Test). * represents extreme outliers.

**Figure 4:**
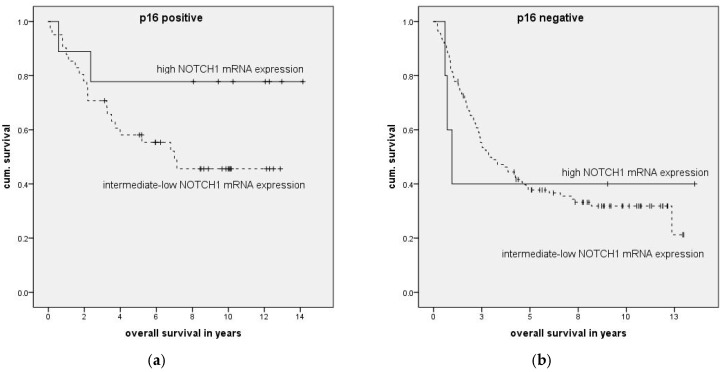
Association of relative NOTCH1 mRNA expression and overall survival in p16-positive (**a**) and -negative patients (**b**) was analyzed by the Kaplan-Meier method followed by log-rank test. A longer overall survival was found for p16-positive patients with relative high mRNA expression of NOTCH1 (**a**) (*n* = 50, *p* = 0.139) but not for p16-negative patients (**b**) (*n* = 118, *p* = 0.967).

Overall, 26% of the patients were p16 positive with most frequent staining in the oropharynx (43%) and least frequent staining in the hypopharynx (8%). Expression of p16 was associated with significantly prolonged overall and disease-free survival (*p* = 0.018 and *p* = 0.008), which was also confirmed in the Cox regression analysis (*p* = 0.001 and *p* = 0.002). The median NOTCH1 mRNA expression was significantly higher in p16-positive patients (median 0.91 vs. median 0.61, *p* < 0.001, Figure 3). A significant correlation was found between p16 staining and NOTCH1 mRNA expression (correlation coefficient: 0.280; *p* = 0.01). Patients with p16-positive tumors with high NOTCH1 expression had longer overall survival (*n* = 50, *p* = 0.139, Figure 4). No noticeable effect was seen in p16-negative patients (*n* = 118, *p* = 0.967, Figure 4).

### 2.4. NOTCH1 Expression in HNSCC Cell Lines

**Figure 5:**
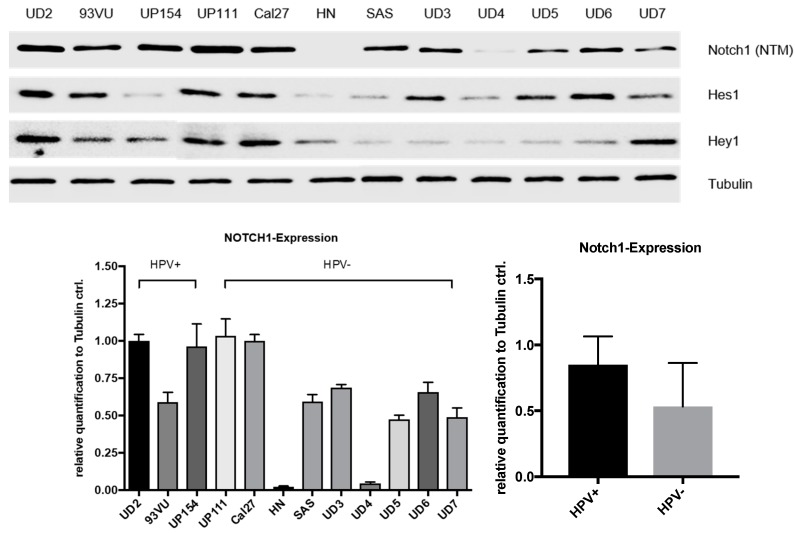
In Western blot analyses, NOTCH1 (NTM) could be detected in all cell lines except for HN (**top**). Quantification revealed higher NOTCH1 protein expression in HPV-positive cell lines compared with in HPV-negative cell lines (*p* = 0.068) (**bottom**). Error bars represent one standard deviation.

HPV16 status of cell lines was confirmed by measuring viral oncogenes E6 and E7 with RT-PCR (Figure S1). NOTCH1 signaling was characterized in three HPV-positive (UD-SCC-2, UP-SCC-154, 93VU) and nine HPV-negative (UD-SCC-3, -4, -5, -6, -7, UP-SCC-111, HN, Cal27, and SAS) HNSCC cell lines with Western blotting (Figure 5). All cell lines except for HN expressed NOTCH1. NOTCH1 (NTM) protein expression was higher in the HPV-positive cell lines compared with in the HPV-negative cell lines, but this was not statistically significant (*p* = 0.068). The NOTCH1, HES1, and HEY1 expression varied considerably in the HPV-negative cell lines.

## 3. Discussion

### 3.1. Expression Analysis

A significant downregulation of NOTCH1, 3, and HES1 was found in comparison with normal tissue. Other researchers have found dissimilar results: in a cohort study of 44 patients with HNSCC, researchers found an upregulation of NOTCH3, HES1, HEY1, JAG1, and JAG2 on the mRNA level in comparison with noncancerous soft palate tissue [10]. The differing results could be due to the different location of normal tissue collected and also due to the smaller sample size analyzed. We analyzed normal mucosa from the oral cavity, oropharynx, hypopharynx, and larynx, which were excised during panendoscopy in proportion to the HNSCC tumor location in our cohort.

### 3.2. Overall Survival Analysis

Subsequently, the association of the differential mRNA expression with clinical outcome was examined. Patients were grouped into high and low expression cohorts (cut-off one standard deviation above or below mean). The subgroup with high NOTCH1 expression had significantly longer overall survival and disease-free survival. Corresponding to this result, patients with high expression of downstream effector HEY1 showed significantly longer DFS. Both results were confirmed in a multivariate Cox regression. As of yet, the role of the expression of key signaling components in HNSCC has only been examined in small patient cohorts or subgroups and differing results have been reported. Consistent with these results, in oropharyngeal squamous cell carcinoma patients, NOTCH1 staining correlated with improved survival [15]. Furthermore, negative staining for NOTCH1 intracellular domain in HNSCC tumors was associated with less differentiation [16]. However, in two reports, HNSCC patients with high NOTCH1 protein expression in immunohistochemistry showed poor prognosis [17,18]. Zhang et al. described higher expression of NOTCH1 and JAG1 in lymph node metastasis-positive tongue cancer [19]. Varying mutation rates and forms of NOTCH1 could explain the discrepancy between immunohistochemistry and quantitative real-time PCR. Mutation rates between 9% to 15% in Western cohorts and of 43% in a Chinese cohort were detected [6,11,12,13]. Predominantly inactivating mutations were reported in Western cohorts whereas a substantial proportion of probable oncogenic mutations were detected in the Chinese cohort [6,11,12,13]. Mutant receptors could, for example, be less degraded and therefore induce higher protein level measurements or truncated receptors to not be expressed at all, so that no protein can be detected. Moreover, NOTCH1 receptor mutation can alter downstream activation significantly, and downstream alterations can also change the effect of NOTCH signaling. Sun et al. reported that patients with mutant NOTCH1 receptor expressed downstream HES1/HEY1 similar to normal epithelium but a large subset of NOTCH1 wildtype patients showed an overexpression of HES1/HEY1 [10]. The proposed bimodal pattern of activation and suppression of NOTCH signaling in HNSCC could also contribute to seemingly contradictory results.

### 3.3. Association of NOTCH1 Expression, p16 Status, and Survival

Significantly higher NOTCH1 mRNA expression was found in p16-positive tumor probes. Additionally, a significant correlation between NOTCH1 mRNA expression and p16 staining was detected. Both high NOTCH1 expression and p16 staining were independent significant factors in the multiple stepwise Cox regression analysis for overall survival. A nonsignificant association was found between patients with high NOTCH1 expression and longer overall survival in the p16-positive patient group (*n* = 50, *p* = 0.139), but not in the p16-negative group (*n* = 118, *p* = 0.967). Unfortunately, p16 immunohistochemistry was only possible in 168 of 195 patient probes due to the consumption of formalin-fixed and paraffin-embedded (FFPE) material in the RNA isolation. The p16-positive group was therefore possibly too small to reach significance. High NOTCH1 expression is therefore probably an independent positive prognostic factor in p16-positive patients only. The differing expression and clinical relevance in p16-positive and -negative tumors could explain part of the above-described discrepancies on the role of NOTCH1 as a tumor suppressor or inhibitor. Since patients with p16-positive tumors are on average younger and healthier, long-term treatment side effects are increasingly becoming relevant. After validation in a bigger p16-positive patient cohort, high NOTCH1 expression could be further analyzed for use as a predictive marker and potentially used, e.g., in future de-escalation studies in p16-positive patients. In line with our results, the immunohistochemical examination of cleaved NOTCH1 expression revealed that negative staining HNSCC tumors were less likely to be HPV-positive [16]. Conversely, Troy et al. detected no correlation between NOTCH1 staining and HPV status in a small cohort of 27 HPV-positive and 40 HPV-negative HNSCCs [20]. This may be due to the small cohort size, disallowing the detection of a correlation. Additionally, protein staining could differ from RNA expression due to receptor degradation.

### 3.4. NOTCH1 Expression In Vitro

Since different NOTCH1 expression levels were detected in p16-positive and -negative HNSCC patients and high NOTCH1 expression was only a positive prognostic factor in p16-positive patients, NOTCH1 signaling was analyzed in three HPV-positive and nine HPV-negative HNSCC cell lines. Corresponding to the higher NOTCH1 expression in p16-positive patient tissues, we also found a trend for higher NOTCH1 expression in the HPV-positive cell lines. NOTCH1 expression greatly varied in the HPV-negative cell lines. There are only limited published data available on NOTCH1 expression in the examined HNSCC cells. Pickering also analyzed NOTCH1 expression in different HNSCC cell lines and detected an association between NOTCH1 mutational status and NOTCH1 expression [6]. Interestingly, NOTCH1 mutations were predominantly found in HPV-negative patient tissue in another investigation [12]. Higher NOTCH1 expression in HPV-positive patients could therefore be due to NOTCH1 wildtype expression. Moreover, it has been reported that cutaneous HPV E6 proteins inactivate NOTCH1 signaling downstream via interaction with mastermind-like (MAML) [21] and thus promotes dedifferentiation. HPV may also inactivate NOTCH1 signaling downstream in HNSCC, explaining the higher expression in p16-positive tumors.

In summary, this is to our knowledge the largest analysis towards determining the clinical relevance of the expression of NOTCH1 signaling components in HNSCC patients. Large alterations in the expression of NOTCH1, 3, and HES1 were detected in comparison with normal tissue. Patients with high expression of NOTCH1 showed significantly better prognosis. The favorable prognostic relevance of high NOTCH1 expression was only seen in p16-positive patients. High NOTCH1 expression should therefore be further evaluated as a prognostic marker in a larger cohort of p16-positive patients. There was also a trend seen for higher NOTCH1 expression in HPV-positive cell lines as compared to HPV-negative cells. Inhibitors of NOTCH signaling are already in clinical testing in other malignancies such as pancreatic and small-cell lung cancers [22,23]. However, NOTCH inhibition seems less promising in HNSCC, since patients with high NOTCH1 expression demonstrated better survival in our study. The findings in this study are therefore highly relevant for the development of NOTCH-targeting therapies in HNSCC.

## 4. Materials and Methods

### 4.1. Patient Tissue Samples

We obtained tissue samples from 195 HNSCC Patients (163 males, 32 females; median age 59 years, range 35 to 89 years) diagnosed between January 2002 and December 2005. As a control group we used mucosa obtained through panendoscopy or tonsillectomy (*n* = 30, 18 males, 12 females; median age 51 years, range 25 to 87 years) from oropharynx (*n* = 7), hypopharynx (*n* = 17), and larynx (*n* = 6). All patients were treated in the Department of Otorhinolaryngology at Klinikum rechts der Isar, Technical University of Munich. Sixty-five patients of the cohort have been used for a previous study [9]. All tissue samples were formalin fixed and paraffin embedded (FFPE). Only one patient received neoadjuvant therapy; all remaining specimens were retrieved before adjuvant or primary radiochemotherapy. The independent ethics committee of the Technical University of Munich approved the study, under project number 1420/05 (13 June 2014) and 107/15 (12 March 2015).

### 4.2.Clinical Data

Clinical data provided by the Munich Cancer Registry were verified with data gathered from filed and electronical medical records. Thirty-nine patients (20.0%) were treated solely with surgery; 95 patients (48.7%) underwent surgical resection combined with radio(-chemo)therapy. Primary radiation or radio-chemotherapy was used in 48 patients (24.6%); 5 patients (2.6%) received palliative radio- and/or chemotherapy. In 8 cases (4.1%), no treatment was applied or treatment could not be reproduced. The median survival calculated by Kaplan-Meier analysis was 4.24 years (min–max follow up period 0.09–14.14 years); the overall five-year survival rate was 46.6%.

### 4.3. RNA Isolation and cDNA Synthesis

FFPE tissue samples were deparaffinized and digested using 40 µL Proteinase K (Roche Diagnostics GmbH, Unterhaching, Germany) in 100 µL PK buffer (50 mM Tris, 1 mM EDTA, and 25% Tween 20 diluted in water) added to 16 µL 10% SDS (10 g Sodiumdodecylsulfat diluted in 100 mL water) at 55 °C. After 24 h, 10 µL Proteinase K was added again and samples were incubated for another day. Further processing was performed using an InviTrap^®^ RNA Mini Kit (Stratec, Birkenfeld, Germany) according to the manufacturer’s protocol. RNA from HNSCC cells was isolated with the RNeasy-Mini-Kit (Qiagen, Hilden, Germany). After isolation the RNA concentration was quantified using the NanoDrop 1000 system (PEQLAB, Erlangen, Germany). We used only probes with a minimal RNA concentration of 10 ng/µL. Afterwards, probes were diluted to a concentration of 10 or 25 ng/µL depending on the initial concentration of RNA and stored at −20 °C. cDNA synthesis was performed using Maxima^®^ reverse transcriptase (Fermentas, Waltham, MA, USA) according to the manufacturer’s protocol.

### 4.4. Quantitative Real-Time PCR

Quantitative real-time PCR (qPCR) was performed to quantify mRNA expression of NOTCH1, NOTCH3, HES1, HEY1, JAG1, HPV16 E6, and HPV16 E7. For normalization of expression levels, GAPDH was used per sample. For qPCR mix, 50 ng cDNA template was added to 12.5 µL KAPA-SYBR Fast Universal (PeqLab, Erlangen, Germany) and 0.5 µL of 20 pmol of each primer. Water was added to a final volume of 25 µL. Primer sequences and specific annealing temperatures are depicted in Table 5. NOTCH1, NOTCH3, HES1, and HEY1 primers were newly designed and GAPDH, JAG1, E6, and E7 primers were used as previously described [7,24,25,26]. After normalization, the ΔΔ*C*t method was used to compare relative expression for NOTCH1 pathway components and gel electrophoresis for HPV E6 and E7.

### 4.5. Immunohistochemical Study

To identify carcinomas associated with HPV infection, p16 expression was analyzed in tumor tissue. From previously identified FFPE blocks, 1.5 µm sections (cut with Microm HM 355 S (International GmbH, Walldorf, Germany)) were placed on glass slides, dewaxed, and rehydrated. Microwave oven heating in citrate-buffered saline was used for antigen retrieval, as recommended by the manufacturer. After cooling, the slides were incubated with the antibody. A CINtec^®^ Histology Kit (Roche Diagnostics GmbH, Mannheim, Germany) containing mouse monoclonal anti-p16INK4a (E6H4) at concentration 1 µg/ml was used according to the manufacturer’s protocol. Tissue with known expression of p16 was used as a positive control.

P16 expression level was described using a scoring system including staining intensity and percentage of stained tumor cells (Table 6). HPV positivity was considered at 3 or more points.

### 4.6. Cell Culture

The Cal27, HN, and UP-SCC-154 cell lines were obtained from DSMZ (Braunschweig, Germany), the UD-SCC-2-7 cell lines were obtained from the University of Düsseldorf (Department of Otorhinolaryngology, Düsseldorf, Germany), the 93VU cell line from VU University Medical Center Amsterdam (Department of Clinical Genetics, Amsterdam, Netherlands), and SAS from JCRB cell bank (Osaka, Japan). All cell lines have been STR profiled and were routinely prophylactically treated against mycoplasma infection. The cells were cultured in Dulbecco’s Modified Eagle Medium (DMEM) (Invitrogen, Darmstadt, Germany) containing 10% fetal bovine serum (FBS) (Biochrom, Berlin, Germany), 2 mM glutamine, 100 µg/mL streptomycin, and 100 U/mL penicillin (Biochrom), maintained at 37 °C in an atmosphere of 5% CO_2_, and grown to 70–90% confluence.

### 4.7. Western Blot Analysis

For protein analysis, cells were grown to 70% confluence in 10 cm tissue culture dishes. Cells were washed with ice-cold 1× DPBS and lysed with 500 μL of cell lysis buffer. The buffer contained 1× Cell Lysis Buffer (Cell Signaling, Danvers, MA, USA), 1 mM PMSF (Carl Roth, Karlsruhe, Germany), and 1× Protease Inhibitory Cocktail (Cell Signaling). The lysis buffer (10×) (Cell Signaling) included 20 mM Tris-HCl (pH 7.5), 150 mM NaCl, 1 mM Na_2_EDTA, 1 mM EGTA, 1% Triton, 2.5 mM sodium pyrophosphate, 1 mM β-glycerophosphate, 1 mM Na_3_VO_4_, and 1 µg/mL leupeptin. Next, the cells were scraped off the culture dishes, pipetted into 1.5 mL microtubes, incubated on ice, and centrifuged at 4 °C and 10,000 rpm for 15 min to isolate the soluble protein fraction. The clarified lysate was frozen at −20 °C until use in the Bradford assay. The Bradford assay was used to verify that equal amounts were loaded per lane on an SDS-PAGE.

Equal protein concentrations (15 μg) were separated for 3 h at 120 V using an SDS-PAGE (Blotting System Mini-PROTEAN^®^ Tetra System and PowerPac^TM^ HC from Bio-Rad Laboratories, Munich, Germany) in a Tris-glycine running buffer. The densities of the running gels ranged from 7.5% to 12.5%, and the stacking gels possessed a density of 5%. The proteins were then transferred to a polyvinylidene fluoride (PVDF) membrane (Merck Millipore, Darmstadt, Germany) using a Trans-Blot^®^ SD Semi Dry Transfer Cell (Bio-Rad Laboratories, Munich, Germany) at 225 mA for 80 min. A solution containing 5% nonfat dry milk in 1× TBS and 0.1% Tween-20 was used to block unspecific binding sites. The membranes were then incubated with the primary antibodies against NOTCH1 (NTM), HES1, HEY1, and Tubulin (all from Cell Signaling Technologies, Danvers, MA, USA) in 1× TBS + 0.1% Tween-20 for 12 h at 4 °C, washed, and incubated with an HRP-linked secondary antibody (Cell Signaling technologies) in 5% nonfat dry milk in 1× TBS and 0.1% Tween-20 for 1 h at room temperature. Next, the membranes were washed and incubated in Thermo Scientific™ Pierce™ ECL Western Blotting Substrate (Fisher Scientific, Waltham, MA, USA) for 1 minute. Immunoreactivity was visualized by ChemiDoc XRS+ with Image Lab^TM^ Software (Bio-Rad Laboratories, Munich, Germany). Protein expression was quantified with scanning densitometry and values normalized to a tubulin control.

### 4.8. Statistical Analysis

All statistical tests were two-sided and significance was determined at a level of 5%. For comparison of mRNA expression in normal tissue versus tumor tissue and p16-positive and p16-negative tissue, Mann–Whitney U Test or Kruskal–Wallis Test was used. Correlation was calculated according to Spearman Rho. Expression in Western blots was compared with *t*-test.

To examine the impact of mRNA expression on clinical parameters we categorized patients into high and low expression groups and compared these to the remaining patients. High and low mRNA expression was defined as mean relative mRNA expression plus or minus one standard deviation.

Association of clinical parameters and high NOTCH1 expression was compared with Fisher’s exact test with Bonferroni correction applied. The impact of expression levels on survival was analyzed with Kaplan–Meier curves, and significance was calculated using log-rank testing. To examine the association of expression levels with clinical data, multivariate forward stepwise Cox regression was performed. Statistical calculations were done in SPSS version 23 (IBM, Ehningen, Germany) or GraphPad Prism 6.0 (GraphPad Software, La Jolla, CA, USA).

## Figures and Tables

**Table 1:** Depiction of the clinical and pathological characteristics of the 195 head and neck squamous cell carcinoma (HNSCC) patients included in this study and association with NOTCH1 expression.

Clinical Characteristics	Overall	NOTCH1 Expression		*p* Value (Fisher Exact)
		Intermediate–Low	High	
**Overall**	**195**	**175 (89.7%)**	**20 (10.3%)**	
Primary site				1
Oral cavity	32 (16.4%)	30 (17.1%)	2 (10.0%)	
Oropharynx	83 (42.6%)	70 (40.0%)	13 (65.0%)	
Larynx	42 (21.5%)	40 (22.9%)	2 (10%)	
Hypopharynx	38 (19.5%)	35 (20.0%)	3 (15.0%)	
Alcohol consumption				0.128
Daily	153 (78.5%)	142 (85.5%)	11 (61.1%)	
Rare/never	31 (15.9%)	24 (14.5%)	7 (38.9%)	
Unknown	11 (5.6%)			
Tobacco exposure				0.072
Smoker	166 (85.1%)	154 (90.6%)	12 (66.7%)	
Nonsmoker	22 (11.3%)	16 (9.4%)	6 (33.3%)	
Unknown	7 (3.6%)			
**Staging and Grading**				
Tumor stage (pathological)				0.096
T1	46 (23.6%)	38 (21.7%)	8 (40.0%)	
T2	53 (27.2%)	44 (25.1%)	9 (45.0%)	
T3	50 (25.6%)	48 (27.4%)	2 (10.0%)	
T4	46 (23.6%)	45 (25.7%)	1 (5%)	
Nodal stage (pathological)				0.032
N0	65 (33.3%)	64 (36.6%)	1 (5.3%)	
N1–3	129 (66.2%)	111 63.4%)	18 (94.7%)	
NX	1 (0.5%)			
Metastasis (initial stage)				1
M0	172 (88.2%)	155 (88.6%)	17 (85%)	
M1	8 (4.1%)	8 (4.6%)	0	
MX	15 (7.7%)	12 (6.9%)	3 (15.0%)	
Grading				1
G1/G2	104 (53.5%)	95 (54.3%)	9 (45%)	
G3/G4	91 (46.7%)	80 (45.7%)	11 (55%)	
p16 Status				0.048
p16 positive	50 (25.6%)	41 (26.6%)	9 (64.3%)	
p16 negative	118 (60.5%)	113 (73.4%)	5 (35.7%)	
p16 unknown	27 (13.8%)			

**Table 2:** Depiction of relative mRNA expression in tumor tissue vs. in normal tissue. Relative expression was compared with the ∆∆*C*t method and *p*-value calculated by Mann–Whitney Test.

Target	Normal Tissue			HNSCC			*p* Value
	Min	Max	Median	Min	Max	Median	
NOTCH1	0.29	2.48	1.15	0.10	5.94	0.69	0.003
NOTCH3	0.41	5.52	0.89	0.11	2.80	0.57	<0.001
HES1	0.33	9.97	0.85	0.16	3.21	0.69	0.049
HEY1	0.26	3.99	0.85	0.01	15.57	1.24	0.254
JAG1	0.18	3.13	1.10	0.19	3.63	0.92	0.270

**Table 3:** Depiction of results of survival analysis with Kaplan–Meier method and log-rank test. Association of relative mRNA expression and overall and disease-free survival. For each target, two comparisons were made: (1) high mRNA expression (mean + 1 standard deviation) vs. intermediate and low expression (remainder); (2) relative low mRNA expression (mean – 1 standard deviation) vs. intermediate and high expression (remainder).

Comparison of Relative mRNA Expression	OS (*p* Value)	DFS (*p* Value)
Comparison of high vs. intermediate—low expression		
NOTCH1	0.013	0.040
NOTCH3	0.568	0.896
HEY1	0.419	0.077
HES1	0.268	0.240
JAG1	0.461	0.481
Comparison of low vs. intermediate—high expression		
NOTCH1	0.611	0.673
NOTCH3	0.633	0.082
HEY1	0.755	0.040
HES1	0.590	0.065
JAG1	0.322	0.529

**Table 4:** Forward stepwise Cox regression with age; sex; T, N, and M status; grading; p16 staining; and high NOTCH1 and high HEY1 expression (mean + 1 SD) as criteria was performed for OS (a) and DFS (b).

**(a) Overall Survival**			
Factor	*p* Value	HR	95% CI
T status	<0.01	1.95	(1.33–2.87)
N status	<0.01	3.29	(2.09–5.18)
Age	0.01	1.03	(1.01–1.05)
p16 positive	<0.01	0.42	(0.26–0.69)
High expression of NOTCH1 (mean + 1 SD)	0.03	0.38	(0.16–0.93)
**(b) Disease-Free Survival—High NOTCH1 and HEY1**			
Factor	*p* Value	HR	95% CI
N status	<0.01	2.60	(1.55–4.35)
M status	0.01	2.38	(1.29–4.38)
p16 positive	<0.01	0.39	(0.21–0.71)
High expression of HEY1 (mean + 1 SD)	0.06	0.41	(0.16–1.02)
High expression of NOTCH1 (mean + 1 SD)	0.03	0.31	(0.11–0.89)

**Table 5:** Primer sequences and specific annealing temperatures used for quantitative real-time PCR.

Primer	Sequence	Annealing Temperature
NOTCH1 forward	TGAATGGCGGGAAGTGTGAAG	62.0 °C
NOTCH1 reverse	GGTTGGGGTCCTGGCATCG	
NOTCH3 forward	ATGGTATCTGCACCAACCTGG	63.0 °C
NOTCH3 reverse	GATGTCCTGATCGCAGGAAGG	
HES1 forward	AAGAAAGATAGCTCGCGGCA	57.0 °C
HES1 reverse	CGGAGGTGCTTCACTGTCAT	
HEY1 forward	CCGACGAGACCGGATCAATA	64.0 °C
HEY1 reverse	GCTTAGCAGATCCCTGCTTCT	
JAG1 forward	ATCGTGCTGCCTTTCAGTTT	56.3 °C
JAG1 reverse	TCAGGTTGAACGGTGTCATT	
HPV16 E6 forward	CAAACCGTTGTGTGATTTGTTAATTA	61.0 °C
HPV16 E6 reverse	GCTTTTTGTCCAGATGTCTTTGC	
HPV16 E7 forward	TTTGCAACCAGAGACAACTGA	58.0 °C
HPV16 E7 reverse	GCCCATTAACAGGTCTTCCA	
GAPDH forward	AGCCACATCGCTCAGACA	56.0 °C
GAPDH reverse	GCCCAATACGACCAAATCC	

**Table 6:** Scoring system used for immunohistochemical identification of HPV-positive carcinomas. Points for staining intensity and staining proportion of tumor cells were summated; HPV positivity was considered at 3 or more points.

Staining Intensity	Points	Staining Proportion	Points
No staining	0	0%	0
Low	1	<10%	1
Moderate	2	10–29%	2
High	3	30–59%	3
		60–100%	4

## Supplementary Materials

Supplementary materials can be found at http://www.mdpi.com/1422-0067/19/3/830/s1.

## Abbreviations

FFPE	Formalin fixed and paraffin embedded
HNSCC	Head and neck squamous cell carcinoma
HPV	Human papilloma virus
OSCC	Oral squamous cell carcinoma
PCR	Polymerase chain reaction
RBPJ	Recombining binding protein suppressor of hairless
