# Supplementary material for: High NOTCH1 mRNA Expression Is Associated with Better Survival in HNSCC
Source: Int J Mol Sci. 2018 Mar 13;19(3):830. doi: 10.3390/ijms19030830 (PMC5877691; doi:10.3390/ijms19030830)
Supplement: Supplementary file 1 [file ijms-19-00830-s001.pptx]

## Slide 1
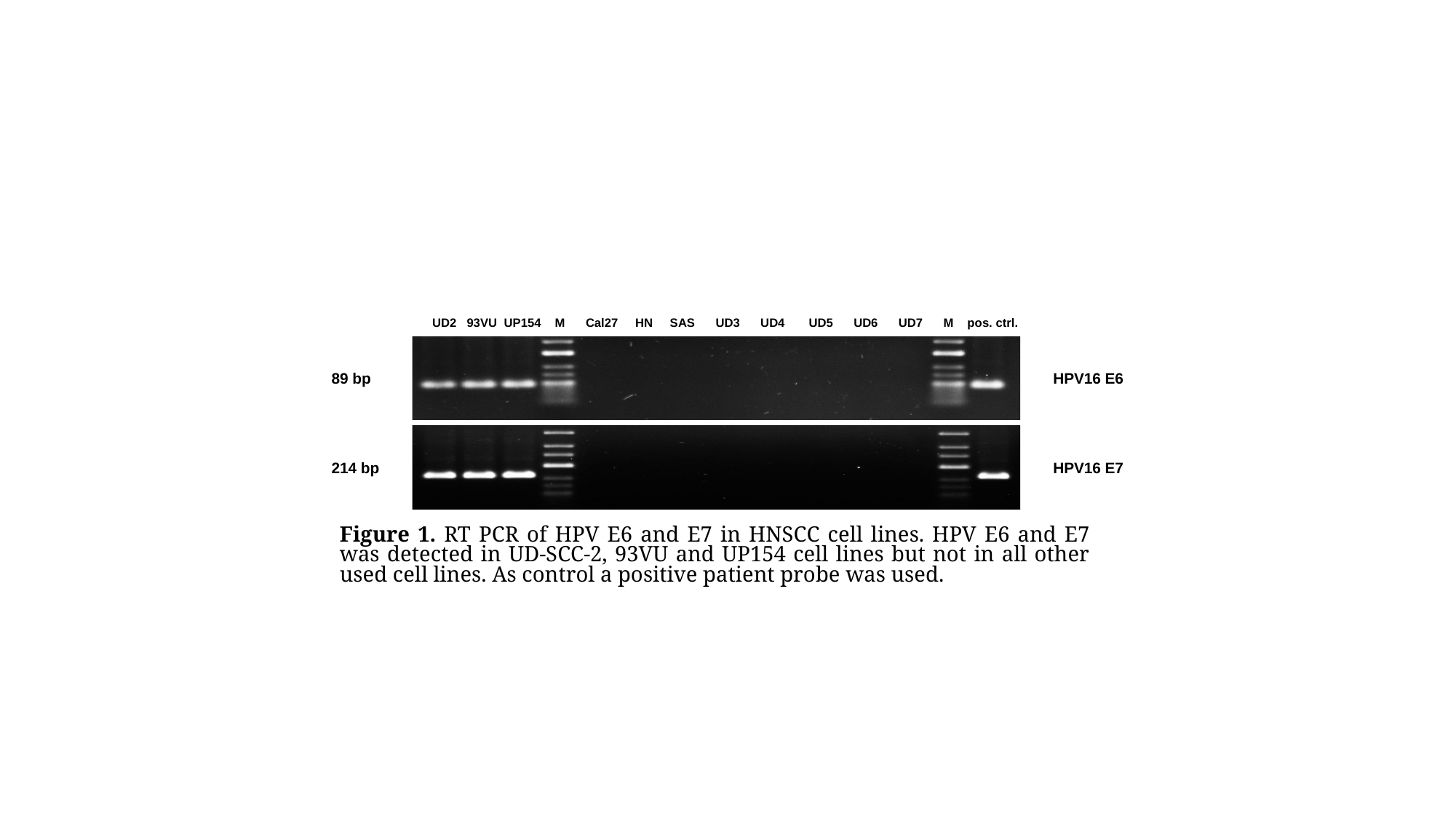

UD2 93VU UP154 M Cal27 HN SAS UD3 UD4 UD5 UD6 UD7 M pos. ctrl.
89 bp
HPV16 E6
214 bp
HPV16 E7
Figure 1. RT PCR of HPV E6 and E7 in HNSCC cell lines. HPV E6 and E7 was detected in UD-SCC-2, 93VU and UP154 cell lines but not in all other used cell lines. As control a positive patient probe was used.
